# The Ah Receptor from Toxicity to Therapeutics: Report from the 5th AHR Meeting at Penn State University, USA, June 2022

**DOI:** 10.3390/ijms24065550

**Published:** 2023-03-14

**Authors:** Gary H. Perdew, Charlotte Esser, Megan Snyder, David H. Sherr, Ellen H. van den Bogaard, Karen McGovern, Pedro M. Fernández-Salguero, Xavier Coumoul, Andrew D. Patterson

**Affiliations:** 1 Department of Veterinary and Biomedical Sciences, Center for Molecular Toxicology and Carcinogenesis, Penn State University, University Park, PA 16802, USA; 2 IUF-Leibniz Research Institute for Environmental Medicine, Auf’m Hennekamp 50, 40225 Düsseldorf, Germany; 3 Department of Environmental Health, Boston University School of Public Health, 72 East Concord Street, Boston, MA 02118, USA; 4 Department of Dermatology, Radboud University Medical Center, P.O. Box 9101, 6500 HB Nijmegen, The Netherlands; 5 Ikena Oncology, Inc., 645 Summer Street Suite 101, Boston, MA 02210, USA; 6 Departamento de Bioquímica y Biología Molecular, Facultad de Ciencias, Universidad de Extremadura, Avenida de Elvas s/n, 06071 Badajoz, Spain; 7 Instituto Universitario de Investigación Biosanitaria de Extremadura (INUBE), Avenida de la Investigación s/n, 06071 Badajoz, Spain; 8 INSERM UMR-S1124, 45 rue des Saints-Peères, 75006 Paris, France

**Keywords:** Ah receptor, aryl hydrocarbon receptor, cancer, clinical trials, OCT4, multiple sclerosis, AHR structure, hematopoiesis, microbiome, immune checkpoint inhibitor, intestinal bowel disease, steatosis, skin barrier

## Abstract

The aryl hydrocarbon receptor (AHR) is a sensor of low-molecular-weight molecule signals that originate from environmental exposures, the microbiome, and host metabolism. Building upon initial studies examining anthropogenic chemical exposures, the list of AHR ligands of microbial, diet, and host metabolism origin continues to grow and has provided important clues as to the function of this enigmatic receptor. The AHR has now been shown to be directly involved in numerous biochemical pathways that influence host homeostasis, chronic disease development, and responses to toxic insults. As this field of study has continued to grow, it has become apparent that the AHR is an important novel target for cancer, metabolic diseases, skin conditions, and autoimmune disease. This meeting attempted to cover the scope of basic and applied research being performed to address possible applications of our basic knowledge of this receptor on therapeutic outcomes.

## 1. Introduction

The aryl hydrocarbon receptor (AHR) is a protein that was first identified in 1976 as a TCDD-binding protein. It was later cloned in 1994 and found to be a member of a family of proteins involved in sensing external stimuli [1]. Initial studies on the AHR focused on its role in xenobiotic metabolism, but subsequent studies in AHR knockout mice revealed its critical role in various physiological pathways, including immune function, barrier function, and reproductive success. A wide-ranging scope of studies from the biochemistry of the AHR, its role in normal physiology, and targeting the AHR for therapeutics were presented at the 5th international meeting in University Park, PA, USA in June 2022. The meeting was organized by Gary H. Perdew and Andrew D. Patterson. The meeting was organized into specific thematic areas that are similarly represented in this report. The AHR field continues to discover new roles for the AHR in biological and disease processes. This information is now being utilized by pharmaceutical companies, with 2022 seeing the first drug, Tapinarof that specifically targets the AHR being approved as a treatment for plaque psoriasis in adults.

## 2. Sessions and Presentations

### 2.1. Biochemistry of the AHR Pathway

**Figure 1:**
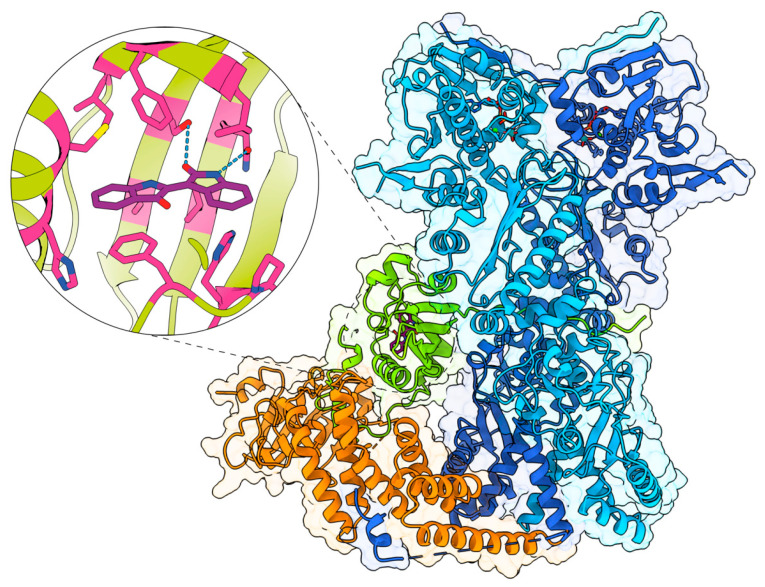
The atomic model of the AHR complex in cartoon representation: HSPA (**light blue**), HSPB (**dark blue**), XAP2 (**orange**), and AHR (**green**, representing amino acid residues 1–437). The insert highlights the ligand-binding pocket with indirubin.

Unliganded cytosolic AHR was found associated with HSP90, XAP2, and p23 [1]. The subunit composition of the AHR was first described using chemical crosslinking as a tetrameric complex composed of the HSP90 dimer, the AHR, and a protein later determined to be XAP2 [2,3]. After the AHR gene was cloned and the ligand-binding domain was defined, there were many attempts to express the ligand-binding domain to obtain an X-ray crystal structure. Purification and isolation of AHR protein for X-ray crystallography failed apparently due to the propensity of the AHR to aggregate upon over-expression. With the advent of cryo-EM that can yield high-resolution structures of protein complexes, this offered a chance to not only illustrate the structure of the core AHR complex but also provide useful ligand-binding pocket resolution. Several months before the actual meeting date, we were approached by William Bourguet from the University of Montpellier, FR, informing the meeting organizers that his research group had successfully obtained a 2.85 Å high-resolution structure of the AHR core complex. To great anticipation, he presented this data in a featured time slot. The cryo-EM structure was accomplished by co-expression of the N-terminal half of the human AHR, HSP90/molybdate, and XAP2, with indirubin in the ligand-binding pocket [4]. A composite map of the AHR core complex is shown in Figure 1. Details of the complex organization reveal that the AHR forms two distinct domains with a linker section that threads through the closed conformation of the hsp90 dimer. XAP2 directly interacts with both hsp90 and AHR consistent with previous biochemical data that revealed that XAP2 stabilized the AHR/HSP90 complex [5]. The resolution of the ligand-binding pocket with perhaps the highest affinity ligand known for the human AHR, indirubin, should provide the appropriate coordinates for docking studies. This information will likely aid the development of different classes of ligands for use in therapeutics.

The AHR binds to dioxin-responsive elements in a variety of gene regulatory regions after heterodimerization with the Ah receptor nuclear translocator (ARNT). However, compared to the AHR, less is known about the regulation of ARNT. ARNT is expressed in most cells as two isoforms that vary in the presence or absence of a 45 amino acid segment [6]. Casey Wright from the University of Texas at Galveston, USA, reported that in human T cells, the level of AHR transcriptional activity is dependent on which isoform of ARNT heterodimerized with the AHR. In addition, the effect of ARNT on NF-kB signaling is also differentially influenced by the proportional expression of each ARNT isoform. The ability of the AHR to heterodimerize with other partners and modulate gene expression was examined by Sudin Bhattacharya from Michigan State University, East Lansing, MI, USA. Results generated with pChIP-seq indicated that KLF6 and RelA are the major dimerization partners of the AHR in the absence of exogenous AHR ligands in the nucleus of tumor cells. Whether these dimerization events are endogenous-ligands-dependent or -independent will need to be explored.

### 2.2. Sources of AHR Ligands and Physiological Outcomes

Exposure to AHR ligands can occur through the diet, microbial metabolism of dietary substrates, and host metabolism. Each of these sources was addressed at the AHR meeting. Broccoli and other cruciferous vegetables contain indologlucosinolates that break down upon consumption to form indole-3-carbinol that in the stomach undergoes acid condensation to form a myriad of products. In particular, indolo [3,2b]carbazole (ICZ), a potent AHR agonist, is formed that is capable of systemically activating the AHR. Studies from the Gary Perdew laboratory revealed that co-feeding parsley and broccoli resulted in greatly enhanced activation of the AHR in various tissues. The proposed mechanism of action relies on the presence of apigenin in parsley, a CYP1A1 substrate, that may compete with the CYP1A1-mediated metabolism of ICZ, increasing the half-life of ICZ. This highlights the potential complexity of a varied diet on AHR activation potential. These studies also examined other whole foods that could modestly increase AHR activation in the intestinal tract. This same laboratory also presented work revealing that broccoli-mediated activation of the AHR decreased small intestinal epithelial cell proliferation and increased goblet cell number. The latter effect appeared to be at least in part through AHR direct transcriptional regulation of the transcription factor Math1. Another aspect of AHR dietary ligand exposure was explored by Heike Weighardt from the University of Bonn, Bonn, Germany, who examined the ability of indole-3-carbinol to support intestinal homeostasis through AHR-dependent and AHR-independent mechanisms [7].

Perhaps, the source of AHR ligands which has received the most attention is from the microbiota and the host to metabolize tryptophan to AHR activators/ligands. Previous studies have determined that relatively high concentrations of tryptophan metabolites that are also AHR ligands are in cecal contents and feces of mice and humans [8]. The dominant tryptophan metabolites from the standpoint of AHR activation at the presented concentration were indole, 2-oxindole, indole-3-acetic acid, and kynurenic acid. The Perdew laboratory presented a poster that examined the concentrations of tryptophan metabolites in human and mouse serum that can activate the AHR at the presented concentrations were kynurenine, kynurenic acid, indole-3-acetic acid, indole-3-propionic acid, indole-3-lactic acid, and indole-3-carboxaldehyde. This list differs from what has been observed in cecal contents. Clearly, which metabolites are from the gut microbiome versus the host will need to be resolved. Studies were also presented examining the therapeutic potential of tryptophan metabolites. Teresa Zelante from the University of Perugia, Perugia, Italy, demonstrated that the administration of the microbial metabolite indole-3-carboxaldehyde in a mouse model of multiple sclerosis was protective through the activation of tryptophan metabolism and the release of serotonin and 5-hydroxytryptamine from mast cells. This response appears to help maintain self-tolerance by expanding regulatory T cells.

### 2.3. AHR and Sphingolipid Metabolism

The AHR is a well-known regulator of lipid metabolism, and recent research has revealed that it also directly regulates several genes involved in sphingolipid metabolism [9]. Sphingolipids, which are important components of cellular architecture and act as signaling molecules, play a role in various diseases, such as obesity and cancer, and in cellular responses, such as apoptosis. Richard Proia from the National Institutes of Health, Bethesda, ML, USA, presented his groundbreaking findings from a genome-wide CRISPR/Cas9 screen, indicating that AHR is essential for sphingolipid levels [10]. That is, *Ahr*-null mice and *AHR* knockouts in HeLa cells exhibit reduced expression of crucial sphingolipid biosynthetic genes. In his poster presentation, Shigetoshi Yokoyama from Penn State University, University Park, Pennsylvania, USA, reported that not only does the AHR impact sphingolipid metabolism, but it may also physically interact with key sphingolipid enzymes. These remarkable presentations suggest that the AHR may act as a central regulator of the sphingolipid pathway.

### 2.4. AHR Toxicology and Metabolism

The role of the AHR in mediating the responses to persistent environmental pollutants such as 2,3,7,8-tetrachlorodibenzo-*p*-dioxin (TCDD) and other halogenated aromatic hydrocarbons is well-established. However, new insights into toxicity continue to arise, particularly with the advent of cutting-edge techniques and the integration of complex data to form a more comprehensive understanding of toxicity mechanisms. The 2022 AHR Symposium emphasized the latest technological advances in metabolomics, single-cell RNA sequencing (scRNA-seq), and microbiome analysis as they offer new perspectives on understanding the effects of environmental chemicals on toxicity and disease.

During his keynote speech, Francisco Quintana from Harvard Medical School, Boston, MA, USA, demonstrated the significance of using integrated systems approaches to discover new factors that contribute to inflammatory bowel disease (IBD) [11]. His team employed a wide range of experimental techniques, including public database mining, animal models, single-cell RNA sequencing, microbiome analysis, and large-scale chemical screening. They proposed that the herbicide propyzamide may play a role in IBD through a pathway involving T and dendritic cells, which is partly controlled by the AHR. Further epidemiological research is needed to confirm these connections, but these intriguing findings will inspire further investigations into the ways in which environmental chemicals can contribute to the development of IBD.

The AHR plays an important role in metabolic diseases, including obesity and fatty liver disease. Rance Nault and Tim Zacharewski from Michigan State University, East Lansing, MI, USA, emphasized the value of sequence-based approaches, including single-nuclei RNA sequencing (snRNA-seq) and spatial transcriptomics [12,13]. They argued that using these methods in combination allows for the spatial resolution of the effects of TCDD exposure on different cell types in the liver, providing a more comprehensive understanding of the pathogenesis of toxicant-induced liver disease. Additionally, they highlighted how incorporating metabolomics with transcriptional profiling can reveal the metabolic consequences of TCDD exposure, specifically on acetyl-CoA and β-hydroxybutyrate production. Additionally, Cornelis Elferink from the University of Texas Medical Branch, Galveston, TX, USA, presented new insights into the role of AHR in regulating fibroblast growth factor 21 (FGF21) and discovered a novel circadian mechanism that affects FGF21 expression and its impact on the metabolism [14]. The data from the Elferink team strongly support the importance of carefully considering the timing of metabolic studies that relate to AHR activation, as circadian clocks can have a significant impact on the results.

Finally, it is increasingly understood that the AHR activation not only affects the microbiome, such as in the gut and on the skin, but also that certain AHR ligands can directly impact the gut microbiome. Yuan Tian and colleagues from Penn State University, University Park, PA, USA, reported that the AHR ligands such as 2,3,7,8-tetrachlorodibenzofuran and polychlorinated biphenyl 126 had a significant impact on the gut microbiome during early life and that they also directly influenced the growth rate and metabolome of key commensal bacteria [15,16,17]. Additionally, Thomas Sutter from the University of Memphis, USA, provided evidence for a connection between AHR activation and the skin microbiome further strengthening the important link between the AHR and microbial partners in and on the host. These findings help to further our understanding of how exposure to environmental chemicals can contribute to negative health outcomes, by providing insight into the mechanisms by which these chemicals exert toxicity through new targets including the microbiome.

### 2.5. AHR and Development

The role of the AHR during development emerged at the end of the last century with the creation of AHR knockout (KO) mice [18,19]. The first results from these models revealed that the AHR played a major role in the development of the liver, the thymus, and the circulatory system. While these results have been confirmed over time, the joint discovery of endogenous ligands, which has accelerated considerably in the last 10 years, and the interest in the role of the AHR in the development of other organs, has greatly expanded the disciplinary field [20].

Chia-I Ko from the College of Medicine, University of Cincinnati, Cincinnati, OH, USA, presented research that underlined the importance of the AHR in the very early stages of embryonic development, notably pre-implantation [21]. More precisely, the AHR activated by endogenous signals plays a crucial role not only in the regulation of embryonic stem cell pluripotency associated with tissue repair but also in the regulation of the phenotype of cancer stem cells. In terms of mechanisms, Chia-I Ko highlighted that the AHR blocks the expression of the “pluripotency” genes Oct4 and Nanog and thus potentiates stem cell differentiation, while limiting cell plasticity. Interestingly, the AHR also regulates blastocyst formation as shown by the decrease in blastocyst numbers in KO models, but also in the case of TCDD treatment, suggesting a competition of this xenobiotic with endogenous ligands. As an illustration, Oct4 is expressed from the “four cells” stage onwards, while AHR expression decreases at this stage of development and practically disappears at the “eight cells” stage [22].

On a related theme, the hematopoietic compartment is complex as both stem cells and highly differentiated cells with all the intermediate pluripotent stages are present. Paige Lawrence from the Department of Environmental Medicine, University of Rochester, Rochester, NY, USA, emphasized the role of AHR in controlling the tempo and tenor of hematopoiesis. She then demonstrated that both the lack of the receptor in hematopoietic stem cells and receptor antagonism led to an increased production of hematopoietic precursors, which may be of therapeutic interest [23]. These disruptions in the AHR functions are also accompanied by changes in the percentages of the erythroid, myeloid, and lymphoid lineages in favor of the myeloid lineage (MPP3) when using either a tamoxifen-inducible model of AHR KO or through the use of an antagonist [24]. The influence of exogenous ligands, such as TCDD, decreases the number of hematopoietic stem cells and, in the long term, induces a decrease in macrophage progenitors. This could influence some anti-viral responses, and more generally, these results echo her work demonstrating the influence of the AHR on the function of T-follicular helper cells during viral infection and the potential role of environmental stressors on induced vulnerability to certain viral infections, such as influenza or severe acute respiratory syndrome coronavirus (SARS) [25]. Given the recent COVID-19 pandemic, understanding the role of AHR in the immune response to such infections and for vaccine efficacy will be both timely and much-needed [26].

Mark Hahn, Biology Department, Woods Hole Oceanographic Institution, Woods Hole, MA, USA, highlighted another key aspect characterizing the AHR through evolution by reviewing the huge difference in sensitivity between species (human, mouse, rat, hamster, guinea pig, invertebrates, etc.) and an element influencing the disruption of its developmental function by exogenous ligands. Mark Hahn described his long-term use of a very sensitive model to prolonged AHR activation, the Atlantic Killifish, in terms of adverse effects on the cardiovascular development [27]. Interestingly, this model identified four genes encoding AHR proteins (namely, 1a, 1b, 2a, and 2b). In areas polluted by polychlorinated biphenyls (PCBs) on the American east coast of the United States, these persistent organic pollutants cause rapid variations in the genetics of killifish populations, particularly in terms of resistance to AHR ligands [28]. Importantly, in addition to the AHR (particularly the isoforms, 1a and 2a), one of the components of the AHR cytoplasmic complex AIP (AHR Interacting Protein) is also involved in this process. The results indicated that AIP has a more prominent role in AHR activation potential in the presence of certain environmental stressors than previously thought.

Overall, these different presentations illustrate the complexity of the AHR regarding its response to our environment, which appears to make this receptor a key responder to the exposome, due to the variable influence of the different ligands capable of modulating receptor functions [29]. It is truly remarkable to see that these ligands can lead to rapid genetic adaptations in sensitive organisms, such as the Killifish, in a relatively short period of time.

### 2.6. AHR and Immunology

Already a decade ago, the identification of the AHR as a link between the environment and T-cell responses fueled enormous interest in the AHR beyond toxicology [30]. It was important to see at the meeting that the role of the AHR in B cells has now also been explored. It is known that immunoglobulin production and secretion are impaired in an AHR-mediated manner. However, given the importance of antibodies in defense against infectious diseases, we still face a significant gap regarding the influence of xenobiotics and possibly beneficial AHR ligands on B-cell responses. Courtney Sulentic from the Wright State University, Detroit, MI, USA, presented novel data demonstrating species differences in the transactivation potential of AHR at the immunoglobulin heavy chain (IgH) locus [31]. The human IgH locus features two ~17 kb transcription control regions located at the 3′end of each Cα gene segment (encodes IgA1 and IgA2). Each control region contains an enhancer with up to four ~53 bp repeats, which are not present in rodents. This enhancer is sensitive to TCDD in an AHR-dependent manner. However, molecular experiments by the Sulentic group suggest that a non-canonical pathway is also active, i.e., the AHR may interact with other transcription factors binding to this enhancer. Additionally, mutation or reduction in the number of ~53 bp repeats differentially affected isotype expression in their Burkitt’s lymphoma cell model, leading to the hypothesis that the human-specific repeats affect sensitivity to AHR ligands, albeit with many unknowns [32]. The current working hypothesis is that varying signals, including infections, influence the transcription-factor-binding profiles, and the ligand-dependent AHR plays a role in fine-tuning antibody production.

In this context, it is important to remember that AHR ligands can also be produced internally. As discussed elsewhere in this report, the availability of ligands from the diet and the gut-residing microbiota is crucial in maintaining a healthy level of AHR-signaling. This was evident from several talks related to the steady-state immune response and homeostasis of certain immune cells. The need for AHR signaling in less well-known immune cells, e.g., such as the need for AHR signaling for the survival of γδ T cells in the skin, was demonstrated by Charlotte Esser from the IUF—Leibniz Research Institute for Environmental Medicine, Germany [33]. Possibly, the role of ARNT and AHRR will become more important here. For instance, Casey Wright examined the interaction of AHR/ARNT isoforms and NF-kB. He found, using transcriptomics and molecular analyses, that a low intracellular ARNT isoform 1:3 ratio enhances AHR responsiveness to ligand activation but abrogates NF-kB-inducing stimuli. Activation of primary CD4^+^ T cells promoted a physiological increase in the ARNT isoform ratio that favors NF-κB signaling, which is further enhanced by suppression of AHR [6]. Karin Loser from the University of Oldenburg, Oldenburg, Germany, presented work on experimental autoimmune encephalomyelitis (EAE), a mouse model mimicking aspects of human multiple sclerosis. Irradiation with UV light reduced EAE severity if completed before triggering the disease by peptide immunization. Langerhans-cell-specific deletion of AHR (but not a T-cell-specific deletion) abrogated this response. In contrast, Karin Loser found also that specific deletion in Langerhans cells protected from disease in a mouse model of systemic lupus erythematosus, a systemic autoimmune disease affecting, among other organs, the skin and the kidney and often triggered by UV irradiation [34]. Accordingly, a better understanding of AHR functions in autoimmune processes is needed, which is the topic of research in a newly established German consortium.

Understanding that viral and bacterial infections are in an “arms race”, as put by Pedro Moura-Alves from the University of Porto, Porto, Portugal, is part of his work investigating AHR function in quorum-sensing of bacteria, i.e., their density-dependent cell-to-cell communication. Quorum sensing is associated with biofilm formation and virulence regulation. The AHR in the eukaryotic hosts has a surprising function to detect quorum-sensing molecules produced by bacteria, e.g., quinolones or phenazines, and thereby possibly orchestrate a host response. As Dr. Moura-Alves showed, the extent of AHR-signaling depends on the differing quorum-sensing molecules produced by bacteria. In other words, the host can spy on the density of the bacterial community and thus influence infection dynamics. The hypothesis is that this would help the host to fight bacterial infections in an adapted and better way.

### 2.7. AHR and Cancer

Some of the most compelling evidence for a role of the AHR in a disease process come from research studying the impact of the AHR on cancer and cancer immunity or, more accurately, cancer immunosuppression. Several papers presented at the meeting provided strong evidence that the AHR plays an important role in several of the classic hallmarks of cancer, including invasion and metastasis, genome instability, resistance to cell death, cell proliferation, altered metabolism, and immunosuppression. As seen in other areas of AHR research, tissue and cellular context as well as the nature of the respective AHR ligands determine whether the AHR drives or limits the disease state. Here, we summarize findings: (1) supporting both pro- and anti-tumorigenic AHR effects in cancer, (2) revealing the contribution of the AHR to suppression of tumor-specific immune responses, and (3) suggesting novel translational strategies to treat cancer through manipulation of AHR function.

#### 2.7.1. Anti- and Pro-Tumorigenic AHR Effects in Cancer

Anti-tumorigenic: Building on a body of previous work [35], Steve Safe from Texas A&M University, College Station, TX, USA, presented new evidence showing that the AHR, driven at least by exogenous ligands, is protective in colon cancer. Using both chemical (azoxymethane) and transgenic (APC^S580/+^; Kras^G12D/+^) murine colon cancer models, these investigators showed that the AHR reduces cancer stem cell formation and cancer initiation, likely through downregulation of the proto-oncogene and cell cycle regulator FoxM1 and through modulation of the function of IL22, a cytokine critical to the integrity of the gut mucosal barrier. Inhibition of FoxM1 activity was then linked to the suppressor of cytokine 3 (SOCS3) and, thereby, the STAT3 signaling [35].

As supported by previous studies showing that AHR depletion in melanoma cells contributes to cancer stem cell senescence [36] and studies on the AHR and “stemness” [37,38,39,40,41], Pedro Fernández-Salguero presented work demonstrating that embryos lacking the AHR exhibited an increase in the stem-cell-associated genes, *NANOG* and *OCT4*. While in the liver, AHR deletion upregulated other mediators of “stemness” including the Hippo-YAP and Wnt/β-Catenin pathways and the pro-inflammatory cytokines TNFA and IL6, thereby resulting in the expansion of OCT4^+^, SOX2^+^, and NANOG^+^ stem-like cells. A similar result was found with lung cells, where AHR deletion upregulated the *K-RasG12D* oncogene and increased stem cell representation. The authors noted that genomic AHR knockout mice are more prone to some cancers including pancreatic cancer, likely because AHR deficiency tends to amplify oncogene-addicted tumors. However, it was not determined if this susceptibility resulted from lost AHR signaling in malignant cells, protective immune cells, or other cells in the tumor microenvironment.

Pro-tumorigenic: Using 2D monolayers and an elegant 3D spheroid culture to model head and neck squamous cell carcinoma (HNSCC), Brandon Yusko from Penn State University, University Park, Pennsylvania, USA, demonstrated that AHR ligands increased while antagonists decreased HNSCC migration. Furthermore, AHR agonists induced inflammatory cytokine production (IL-1, IL-6, and TNF) while increasing metalloproteinase activity, an outcome that likely facilitates malignant cell invasion through basal membranes. Similarly, Toivo Maimet’s laboratory, University of Tartu, Tartu, Estonia, demonstrated that the AHR drives migration in pancreatic ductal adenocarcinomas (PDAs), an extremely aggressive and currently therapeutically intractable cancer. Increased PDA cell migration appeared to be mediated by the AHR-target gene *PTGS2* which encodes the enzyme responsible for the production of PGE2, a known driver of angiogenesis and cell migration [42].

Kanita Chaudhry and Anna Bianchi-Smiraglia from Roswell Park Comprehensive Cancer Center, University of Buffalo, Buffalo, New York, USA, revealed that the AHR drives the *MycN* oncogene and retinoic acid pathways in neuroblastoma increasing clonogenicity and cell invasion. AHR deletion led to neuronal differentiation, potentially driven by increased retinoic acid receptor activity and decreased availability of *MycN*-binding chromatin. The magnitude of this discovery is reflected in part by the observation that nearly half of all high-risk neuroblastomas exhibit *MycN* amplification concomitant with reduced responsiveness to retinoic acid therapy. These results suggest a new route to regulation of *MycN*, long considered to be an undruggable target, through AHR inhibition.

Returning to melanoma, but in this case as an example of AHR pro-tumorigenic activity, Marie Galibert from Rennes University Hospital, Rennes, France, presented data demonstrating that the AHR contributes to BRAF inhibitor (BRAFi) resistance. Indeed, treatment of melanoma cells in vitro with BRAFi increases AHR signaling exemplified by increases in *THBS1*, *TiPARP*, *CYP1B1*, *EGFR*, and *RUNX2*. Notably, mechanisms responsible for these outcomes may reflect interaction of the AHR with Src and a phosphorylation cascade as much as AHR transcriptional activity. Specifically, AHR activation increased Src phosphorylation and signaling resulting in increased cell invasiveness. This serves as a reminder that AHR interaction with other proteins (e.g., Src, RelA, RelB, KLF6, tissue factor, Rb, BMAL, and SPHK2) [43,44,45,46] is understudied [47].

Christiane Opitz, German Cancer Research Center, Heidelberg, Germany, continues to scrutinize metabolic signaling affecting AHR activity, specifically regarding the role of tryptophan-metabolizing enzymes and the AHR ligand production [48,49,50,51]. At this meeting, she presented data using differential gene expression approaches to define an AHR signature. This AHR signature was seen across 32 tumor types and was inversely correlated with patient survival. Importantly, expression of the L-amino oxidase interleukin-4-induced-1 (IL4I1) enzyme strongly correlated with the presence of the AHR signature, more so than the IDO1 dioxygenase, generally thought to be the master regulator of tryptophan metabolism and production of tryptophan-derived AHR ligands. This observation is significant since IL4I1 is a key enzyme in the tryptophan metabolic pathway and has been shown to promote cancer cell mobility and immune suppression [52]. Furthermore, IL4I1 expression correlated with increased tumor infiltration by myeloid-derived suppressor cells (MDSCs) and regulatory T cells (Treg). Conversely, IL4l1 knockout led to decreases in PD1, 41BB, TIGIT, BLIMP1, and CD244 on CD8^+^ T cells. Furthermore, ILFl1 appears to increase with immune checkpoint inhibitor treatment suggesting a metabolic route bypassing IDO blockade and potentially explaining the failure of IDO inhibitors, as monotherapies, to show a significant therapeutic benefit in the clinic.

Snyder and Sherr from Boston University, Boston, MA, USA, provided evidence that the AHR is highly tumorigenic in oral and lung cancers by demonstrating that AHR deletion from aggressive oral or lung cancer cell lines implanted into immunocompetent mice imparted complete or partial immunity, respectively, to challenge with fully malignant AHR-positive tumor cells. In the case of oral cancer cells, injection of AHR-negative tumor cells resulted in 100% immunity corresponding with the acute recruitment of non-exhausted CD4^+^ and CD8^+^ T cells. Interestingly, in the lung cancer model, some AHR-negative tumors eventually grew out providing an opportunity to evaluate if escape variants have exploited compensatory signaling pathways to bypass AHR signaling and to evade cancer immune responses.

In general, at least two non-mutually exclusive mechanisms could account for what seems to be conflicting roles of the AHR in cancer. Initially, the nature of the ligand and AHR co-activators in any given tissue or cell could dictate the spectrum of genes that the AHR regulates and thereby whether AHR activity is anti- or pro-tumorigenic. For example, while tumor-derived endogenous AHR ligands may drive pro-tumorigenic pathways, microbial- or flavonoid-derived ligands may redirect AHR signaling towards other outcomes that limit cancer progression [53]. Alternatively, or in addition, the AHR could drive opposing elements simultaneously, for example, cell growth vs. cell death pathways or robust immunity vs. immunosuppression with the unique cellular or tissue contexts determining which outcome predominates. This may explain why, at least to date, AHR inhibitors are only moderately effective in slowing cancer growth when given alone but work well in combination with immune therapy.

#### 2.7.2. AHR, Immune Checkpoints, and Cancer Immunosuppression

Expanding on their previous work [54], Karen McGovern, Ikena Oncology, Boston, MA, USA, noted that a network of AHR signaling and IDO1/2-mediated production of endogenous tryptophan-derived AHR ligand(s) helps maintain an immunosuppressive tumor microenvironment. These dioxygenases contribute to the production of kynurenine, long known to be an immunosuppressive metabolite and marker for IDO1/2 as metabolic immune checkpoints. Indeed, the authors indicated that IDO1/2, TDO, and CYP1B1 correlate with the presence of immunosuppressive Treg and M2 macrophages in melanoma, non-small-cell squamous lung cancer, and pancreatic cancer. Furthermore, an AHR inhibitor, IK-175, enhanced CD8^+^ T cells and macrophage representation in draining lymph nodes and production of generally immunoprotective cytokines IL2, TNFα, and IFNγ.

Along similar lines, Sherr and colleagues demonstrated that the AHR regulates PD-L1 expression on murine and human oral cancer cells at least in part through direct *Cd274* transcription. Moreover, AHR deletion from oral cancer cells resulted in fewer T cells expressing the T-cell exhaustion markers and mediators of immune checkpoints CTLA4, LAG3, and CD39 and a higher density of activated CD4^+^ and CD8^+^ T cells in tumor-draining lymph nodes. They also demonstrated that signaling through IFNγ, likely produced chronically in the tumor microenvironment, dramatically increases both IDO1 and PD-L1 expression in an AHR-dependent manner implicating a T cell→IFN-γ→AHR→IDO+PD-L1 immune checkpoint amplification pathway in both oral and lung cancers. Their data suggest that the AHR may regulate multiple immune checkpoints during cancer progression.

#### 2.7.3. AHR-Related Novel Translational Strategies for Cancer

Considering the galaxy of AHR-mediated processes in cancer, there are likely many approaches to translate the basic science to the clinic. For example, a relatively simple approach to reducing at least colon cancer risk, as suggested by Steve Safe and colleagues [55], may be through strategic changes in the diet and thereby consumption of dietary AHR ligands. The multitude of studies presented at the conference demonstrating AHR ligand production by the microbiome strongly suggests that modification of at least the gut microbiome may similarly lead to beneficial modulation of AHR activity within immune and malignant cells.

Presentations implicating several potentially compensatory tryptophan-metabolizing, AHR-ligand-generating enzymes (e.g., IDO1/2, TDO, and IL4I1) suggest the limitations of monotherapies targeting any one of these metabolic immune checkpoints. Rather than blocking the production of AHR ligands, several studies suggest the use of AHR inhibitors to block downstream AHR signaling in malignant and/or immune cells. In melanoma, Marie Galibert and colleagues suggested the use of AHR inhibitors in combination with Src inhibitors to bypass the AHR-mediated BRAF inhibitor resistance [56]. AHR-driven HNSCC migration, as exemplified by the studies from the Gary Perdew laboratory, suggests the use of AHR inhibitors to limit tumor invasion and/or metastasis. Remarkably, Chaudhry and Bianchi-Smiraglia discovered that an FDA-approved drug previously used to treat parasitic infections in children, clofazimine, is a putative AHR antagonist that reduced neuroblastoma clonogenicity, invasion, and tumor burden. They suggest that this relatively safe and economical drug be viewed as a potential new therapy for pediatric neuroblastoma patients as a monotherapy or in combination with retinoid therapy. The studies from the David Sherr laboratory demonstrate that novel AHR inhibitors that reduce tumor cell growth can block both PD-L1 expression on tumor cells and prevent T-cell exhaustion, bringing AHR inhibitors into the realm of general immune checkpoint inhibitors. Perhaps, the most advanced efforts to exploit the AHR for human cancer therapy have been made by Karen McGovern and colleagues demonstrating that the AHR inhibitor, IK-175, reduced tumor growth modestly as a monotherapy but more robustly in combination with immune-checkpoint-specific antibody in melanoma and colorectal cancer models. IK-175 is now in phase 1 clinical trials for solid tumors.

Finally, as summarized by Francisco Quintana during the keynote lecture, there are multiple sources of AHR ligands, including the environment, microbiota, diet, and malignant cells themselves. As mentioned earlier, Gary Perdew and colleagues demonstrated that many of these ligands can be found in circulation. Given the long-standing observation that different AHR agonists can affect different transcriptional and functional outcomes, it seems likely that at least some of these ligands have the potential to influence the aggressiveness of transforming cells and the efficacy of anti-tumor responses. With the definition of the 3D structure of the AHR binding site, as reported by William Bourguet, it may now be possible to distinguish between AHR ligands that drive pro- and anti-tumor responses and to more rationally design more potent and effective AHR modulators.

### 2.8. AHR and Skin

The dual role of AHR signaling in the skin being associated with both health-promoting effects and skin disease initiation was poised as “the Janus-faced role” of the AHR in the skin [57]. Albeit the well-studied carcinogenic effects of AHR signaling by environmental pollutants such as dioxins or UV radiation (leading to chloracne, dermatitis-like inflammation, and/or skin cancer), attention in recent years has been directed towards the anti-inflammatory effects and skin barrier reinforcement by AHR activation in chronic inflammatory skin diseases such as psoriasis and atopic dermatitis (or atopic eczema) [58]. The growing body of evidence on its therapeutic effects has culminated into the recent FDA-approved targeted topical therapy in which an AHR agonist, Tapinarof, in ointment formulation is used for local treatment of adult psoriasis patients [59]. Phase 3 trials in atopic dermatitis with Tapinarof are currently ongoing with the prospect of finalization in 2023. This new topical drug may well become the long-awaited alternative to the highly effective, yet user-unfriendly, coal tar therapy. In hindsight, coal tar in fact has been the first topical AHR-targeting treatment in dermatology, yet nowadays considered obsolete. To fully appreciate the efficacy and safety of AHR-targeting therapeutics and considering the emerging market size for the development of novel AHR ligands with enhanced pharmacological properties, researchers should focus to expand our fundamental knowledge on the short- and long-term AHR signaling transduction in various cell types of the skin. In addition, exposure within the (inflammatory) tissue microenvironment, the duration and strength of AHR activation should be considered. Ligand-dependent effects may result from the binding of specific AHR ligands to other keratinocyte receptors, like the EGFR by environmental PAHs, to modulate the outcomes of AHR ligand exposure, as presented by Christian Vogel from IUF—Leibniz Research Institute for Environmental Medicine, Dusseldorf, Germany [60].

Exposome-related modulation of AHR in skin extends to food intake, knowing that many (cruciferous) vegetables are rich sources of AHR dietary ligands. Although most research focuses on local effects in the gut, a poster from the Gary Perdew’s group showed peripheral AHR activation (lung and liver) based on the diet supplementation of mice with specific vegetables known for their AHR-inducing potential (e.g., broccoli). Although no skin data were analyzed in this study, the work presented by Elodie Segura from INSERM, France, exemplifies the presence of a gut–skin axis driven by dietary AHR ligands. In her work, dietary AHR ligands dampened the severity of Th2 responses that drive allergic diseases. These results are in contrast with studies of oral exposure of benzo(a)pyrene in mouse models of acute skin inflammation, where AHR activation potentiated Th2 pro-inflammatory responses [61]. Ligand-dependent effects and the differences in inflammatory processes involved may drive these diverse outcomes. Elodie Segura showed that in a mouse model of cutaneous papain-induced allergy, the lack of dietary AHR ligands impaired Langerhans cell migration to the lymph nodes (potentially through modulation of TGFβ levels) and exaggerated T-cell responses upon papain exposure. The levels of dietary AHR ligands were associated with changes in the transcriptomic landscape of skin keratinocytes, meaning that the presence of AHR ligands in the diet was correlated with a downregulation in keratinocyte response to inflammation. Interestingly, genes involved in barrier function were not altered, while these effects are typically seen for therapeutic exogenous AHR ligands (as presented for coal tar, leflunomide, and SAHRM molecules by Ellen van den Bogaard from the Radboudumc, The Netherlands; or skin-microbiota-derived AHR ligands presented by Thomas Sutter from Memphis University, Memphis, TN, USA). This mouse papain model may not reflect pathophysiological processes that initiate human dermatological diseases, including atopic dermatitis. Yet, the concept of the gut–skin axis being under the control of AHR signaling allows for the speculation that the high rise in allergic and hypersensitive patients with skin manifestations may be due to a general lack of cruciferous vegetables in the Western diet that may negatively influence skin homeostatic processes. Many of the immunity and inflammation-related processes that AHR plays a role in, as discussed in the immunology section, also hold true for the skin. This year’s meeting, however, was much more focused on AHR in the epidermis of the skin, related to the barrier function of skin that appears to be under the (partial) control of AHR signaling.

The AHR-dependent modulation of epithelial barrier function through diet and microbiome-derived metabolites has been investigated extensively in mouse models of the gut [62]. Additionally, for the skin microbiome, studies have indicated the activation of AHR signaling by commensal bacteria and fungi [63,64]. However, whether the microbiota modulates the barrier function remained unanswered. The collaboration between the groups of Thomas Sutter, and Elizabeth Grice, University of Pennsylvania, Philadelphia, PA, USA, yielded the first evidence of such a key controlling function of the skin microbiota in maintaining and restoring the barrier function of skin [65]. Thomas Sutter presented extensive mouse studies including microbiota transplantations in germ-free and/or transgenic mice, by which the team could show that germ-free mice appear to have downregulated transcription levels of epidermal differentiation and cornification-related genes and that commensal skin microbiota present on human skin modulates skin barrier repair mechanisms through keratinocyte-dependent AHR activation in murine skin. These data fitted well with the unpublished data presented by Ellen van den Bogaard on the emerging aspects of AHR signaling in human keratinocytes related to disease in which barrier defects and microbiome dysbiosis are key determinants. In atopic dermatitis, microbiome dysbiosis depletes a specific commensal niche of Gram-positive anaerobic cocci. Using a combination of human 3D skin microbiome models and in vitro host–microbe exposures, it was found that these commensal bacteria produce indoles and provide alarm signals to induce anti-microbial peptide production in keratinocytes and induce expression levels of key skin barrier genes. Herein, only the latter appeared AHR-dependent in studies involving AHR antagonist co-stimulation. While these studies clearly point towards a microbiome-mediated modulation of skin AHR signaling, the actual molecular events that influence the endpoint parameters in the experimental models (e.g., transepidermal water loss, inflammatory infiltrate, and epidermal gene expression levels) remain to be uncovered.

At the molecular level, a complex regulatory network including metabolic reprogramming of glycolysis, sirtuin 1-response, and cellular redox states appears to control epidermal differentiation through pathways including cornified envelope proteins, lipid biosynthesis, and cell adhesion [66]. Thomas Sutter proposed a master regulator role for the nuclear partner of AHR, ARNT, while novel AHR interacting partners in the AP-1 and AP-2 family were discussed by Ellen Van den Bogaard. In contrast to prior beliefs on the putative direct binding of AHR to promotor regions of epidermal differentiation genes, Van den Bogaard’s group found early responsive genes upon AHR ligand stimulation enriched for canonical transcription factors known to promote keratinocyte differentiation, barrier development, and host defense, such as Transcription Factor AP-2α (TFAP2A). In contrast, late AHR-responsive genes were related to epidermal differentiation and structure (e.g., filaggrin, keratins, and transglutaminases) and host defense (e.g., *PI3* and *S100* genes) but were not bound by the AHR in ChIP-seq analysis, in contrast to confirmed AHR binding at the TFAP2A gene locus. Distinct expression patterns of AHR target genes upon AHR activation in the human epidermis corroborates with the notion that physiological terminal differentiation of human keratinocytes is not merely a result from direct transcriptional regulation of terminal differentiation genes but rather involves a multi-step process that is initiated in the stratum spinosum in which the AHR could serve as a key starting point. With more of these omics-data sets emerging in various cell types using various ligands, computational approaches will become a crucial tier in identification and selection of targets for dedicated experimental validation at the lab bench using precious patient samples, laborious experimental models, and refine or reduce animal studies.

### 2.9. AHR and Other Diseases

The implication of AHR in additional pathologies was also discussed during the meeting. Liver-related diseases, not limited to liver cancer, are gaining attention in the field. Thus, we learned from Wen Xie from University of Pittsburgh, Pittsburgh, PA, USA, that the AHR suppresses the activation of hepatic stem cells (HSCs) to limit liver fibrosis in the mouse, a result suggesting that AHR activation by non-toxic ligands in HSCs may be helpful to treat liver fibrosis [67]. AHR also has relevant roles in alcoholic and non-alcoholic fatty liver disease (NAFLD). Aditya Joshi’s group, University of Oklahoma Health Sciences Center, Oklahoma City, OH, USA, presented data indicating that the tryptophan metabolite cinnabarinic acid (CA) activates the hormone stanniocalcin (Stc2) and confers cytoprotection against different stresses in an AHR-dependent manner; consequently, CA may have hepatoprotective roles against NAFLD through AHR [68]. CA also has cytoprotection against steatosis and hepatic damage in an Stc2-dependent manner in alcoholic liver disease mouse models by modulating ERK1/2 activity. Overall, these studies underline the existence of a CA-AHR-STC2 signaling cascade relevant in those liver diseases [69].

AHR was also examined in the context of neurodegenerative diseases. Guillermo Elizondo, from Cinvestav-IPN, Mexico City, Mexico, revealed that AHR activation upregulates the expression of the Parkin gene (*Prnk*) in the mouse ventral midbrain and in human neuroblastoma cells, in which a neuroprotective effect against rotenone neurotoxicity was achieved; these results led to the conclusion that Parkin induction by non-toxic AHR agonists could be a strategy to delay the onset of Parkinson’s disease [70]. On the other hand, Emmanuel Ojo and Shelley Tischkau from Southern Illinois School of Medicine, Springfield, IL, USA, set up primary mouse hippocampal astrocytes and neuronal cell cultures to analyze the impact of AHR activation in the inflammatory processes associated with Alzheimer’s disease. Preliminary results indicated that those culture models could be in fact useful to analyze the role of the AHR in Alzheimer’s pathology.

The AHR and inflammation were also discussed from different perspectives with respect to the lungs. Carolyn Baglole´s group from McGill University, Montreal, QC, Canada, showed that cannabis smoke exposure activates AHR in the lungs and that, in the absence of receptor, cannabis smoke can promote inflammation by increasing monocytes and neutrophils numbers possibly associated with the upregulation of cannabinoid-metabolizing enzymes. Thus, the AHR may have a pivotal role in controlling cannabis-induced pulmonary inflammation. AHR also protects against airway inflammation by controlling autophagy, as reported by Peisong Gao from John Hopkins School of Medicine, Baltimore, ML, USA [71]. Using AHR epithelial conditional null mice they found that, in type II alveolar epithelial cells (AT2), AHR deletion exacerbated the allergen-induced inflammatory response concomitantly to an increase in autophagy. As a conclusion, AHR expressed in AT2 cells may represent a protective mechanism to ameliorate allergic airway inflammation by controlling autophagy.

The autoimmune thyroid eye disease (TED) was addressed in the context of AHR by Collynn Woeller’s group from the University of Rochester, Rochester, NY, USA. Insulin-like growth factor 1 receptor (IGF1R) signaling mediates the activation of orbital fibroblasts and TED pathology. These authors showed that AHR levels are reduced by IGF1R in orbital fibroblasts and that AHR activation impaired IGF1R-induced orbital fibroblasts proliferation and migration. The data presented suggest that AHR loss results in IGF1R-dependent fibroblasts activation in TED and that AHR activation could be a potential therapeutic approach in this pathology.

### 2.10. AHR and Therapeutics

Given the extensive role of AHR in innate and adaptive immunity, it is not surprising that AHR modulation has promising therapeutic potential for many different diseases. The use of AHR antagonists in cancer patients to reverse immune suppression and of AHR agonists in autoimmune disorders to overcome chronic inflammation is widely supported based on years of research on the activities of AHR in multiple cell types. Karen McGovern described the characterization of the AHR antagonist IK-175, a novel orally bioavailable small molecule that is being developed as a potential anti-cancer drug [72]. IK-175 potently inhibits AHR in cell types of multiple species and in in vivo models, including on-target inhibition of AHR-dependent gene expression. IK-175 can inhibit tumor growth in CT26 and B16-IDO mouse tumor models as a single agent and when combined with anti-PD-1 antibodies increases the percentage of complete responses. In addition, IK-175 affects the tumor microenvironment leading to more activated immune cells with an increase in pro-inflammatory cytokines. This immunostimulatory effect of AHR inhibition observed in mouse models was also seen in human T cells, where IK-175 led to a decrease in IL-22 and an increase in IL-2. Studies utilizing a signature of AHR-regulated genes and an immunohistochemistry method for assessing nuclear AHR in human tumors pointed to bladder cancer and head and neck cancer as tumor types, where AHR may play a major role. A phase 1a/b open-label study of IK-175, alone and in combination with nivolumab in patients with locally advanced or metastatic solid tumors and urothelial carcinoma, is in progress (clinicaltrials.gov NCT04200963).

AHR activation may provide a benefit for patients by increasing immune suppression in autoimmune diseases, as has been demonstrated with multiple indoles [73]. Luigina Romani from the University of Perugia, Perugia, Italy, presented data on the microbial metabolite indole-3-aldehyde (3-IAld) that has good activity as an anti-inflammatory via AHR [74,75]. Luigina Romani presented compelling data with 3-IAld in the DSS mouse model of inflammatory bowel disease, where 3-IAld repairs the colon damage and improves the epithelial barrier integrity via AHR and induction of IL22. 3-IAld was also able to protect against immune checkpoint inhibitor (ICI)-induced colitis, while not blocking the anti-tumor effect of ICI [76]. In addition, the Romani group formulated 3-IAld in microparticles for localized delivery and demonstrated remarkable anti-inflammatory activity in a cystic fibrosis model after a single inhalation, with no systemic exposure in the animals. These data suggest that the AHR-ligand activity of 3-IAld could move forward for development as an active compound for the treatment of human diseases.

## 3. Conclusions

The body of data presented in this meeting provided evidence that the AHR is a genuine therapeutic immune target. Targets beyond TNF for anti-inflammatory auto-immune treatments are on the horizon, and activating AHR has shown the potential to be a differentiated and tolerable treatment, especially given the lack of systemic exposure seen preclinically to date. The potential for AHR antagonism in oncology is broad, with immune-oncology field looking for a more durable impact on the tumor microenvironment and differentiation from and synergy with checkpoint mechanisms. AHR activity in multiple cell types in the tumor microenvironment coupled with the general tolerability seen in early studies suggests that it could be a valuable addition to the treatment paradigm in immune-oncology.
